# Foramen of Winslow hernia years after a Ladd’s procedure: A case report

**DOI:** 10.1016/j.ijscr.2020.06.091

**Published:** 2020-06-25

**Authors:** Shadi Abu-Swis, Nir Cohen, Nadav Wallach, Adam Abo-Sharb, Gilbert Sebbag, Waleed Kian

**Affiliations:** a Department of General Surgery, Soroka University Medical Center and Ben Gurion University, Beer Sheva, Israel; b Faculty of Health Sciences, Ben-Gurion University of the Negev, Israel

**Keywords:** Case report, Internal hernia, Foramen of Winslow hernia, LADD”s procedure

## Abstract

Foramen of Winslow hernias form 8% of all internal hernias. They present with non-specific findings and are often diagnosed late in disease progression. Delayed diagnosis of the hernia is associated with an estimated mortality of 50%. This rare event has yet to be described in the literature as a follow-up complication to a Ladd’s procedure.

Here, we present a young male patient with a surgical history of a Ladd’s procedure with a chief complaint of an acute-onset, severe abdominal pain accompanied by episodes of emesis. Prompt clinical analysis, imaging and fluid resuscitation was conducted. A computed tomography (CT) scan showed a mesenteroaxial gastric volvulus and air was identified within the Falciform Ligament. Consequently, the patient underwent an exploratory laparotomy and the foramen of Winslow hernia was identified. An excision and anastomosis procedure was performed, and the cecum was fixed in the lower left quadrant. The procedure had no complications and the patient was discharged from the hospital on the fifth post-operative day.

The purpose of this case report is to present an unusual patient who experienced a foramen of Winslow hernia involving the small bowel six years following a Ladd's procedure. While a causative relation cannot be made between these two events, we theorize that gastrointestinal hypermobility, being one of the three properties leading to herniation, played a role in both pathologies. Therefore, a vigilant surgeon should keep in mind that intra-abdominal congenital malrotation can put patients at greater risk for future herniation.

## 1 Introduction

Internal hernias are caused by an abnormal protrusion of an organ through a congenital or acquired mesenteric or peritoneal opening. The pathophysiological mechanism is believed to be due to a coexistence of increased mobility of small or large bowel, increased intra-abdominal pressure and an enlargement of an anatomical opening; such as the foramen of Winslow. Foramen of Winslow hernias are a rare type of internal hernia and accounts for 8% of internal hernias and 0.08 % of all hernias [1,2]. The borders of the hernia include the caudate lobe superiorly, the duodenum inferiorly, the inferior vena cava posteriorly and the hepatogastric ligament anteriorly [3]. Herniation occurs under the lesser omentum free edge and the hepatoduodenal ligament. The content of the hernia is small bowel in 63 % of cases, the transverse colon accounts for 7% and in less likely circumstances the ascending colon and cecum can herniate as well [4]. There is a broad spectrum to how patients present in such an event. They may indeed present with an acute abdomen, however, their clinical picture can range from nonspecific abdominal pain through to symptoms of obstruction. Consequently, this makes a diagnosis of a foramen of Winslow hernia challenging [5]. A thorough anamnesis may reveal a history of chronic or recurrent abdominal pain before an acute presentation. This is likely due to spontaneous episodes of reduction of the hernia. Early diagnosis is crucial, since late intervention is associated with a mortality rate of approximately 50 %. The rapid clinical deterioration is due to strangulation and subsequent necrosis of the incarcerated bowel segment [6].

Currently, a computed tomography (CT) plays a pivotal role in diagnosis of patients suffering from the foramen of Winslow hernia. Not only does it provide an accurate diagnosis, but the added information can better inform the severity of the incarceration or strangulation. This occurs by showing evidence of damage to the intestinal wall causing pneumoperitoneum [7]. Exploratory laparoscopy or laparotomy have the greatest sensitivity and specificity, saving valuable time in patients presenting with abdominal peritonitis on physical examination. Lastly, the use of plane abdominal X-ray or barium contrast examination is rarely used due to low sensitivity and specificity. However, it may be reserved in cases where small bowel obstruction is considered in the differential diagnosis. Moreover, it is an important tool in places where CT imaging is not available. The mainstay of treatment requires an urgent surgical approach aiming at reduction of the herniated content [8]. Surgical resection is a last resort in cases of irreversible ischemia. In this case report, we present a patient who had a foramen of Winslow hernia involving the small bowel several years following a Ladd's procedure.

This report was written in accordance with the SCARE guidelines [9].

## 2 Case report

A 20-year-old male of Middle Eastern descent, with a surgical history of a Ladd's procedure, walked into the emergency room complaining of severe diffuse abdominal pain and multiple episodes of emesis starting 24 prior to admission. The otherwise healthy patient had a Ladd's procedure six years prior due to intestinal malrotation. The patient denies the passing of stool or flatulence in the day prior to his admission and did not have a fever. On physical examination, the vital signs were normal, the abdomen was non-distended, peristalsis was weak on auscultation and pain was noted on palpation in the right upper quadrant. There were no signs of peritonitis.

At this stage, the differential diagnosis included small bowel obstruction due to adhesions, a perforated gastrointestinal ulcer and an internal hernia. A nil per os (NPO) laboratory test and an abdominal X-ray were ordered. IV fluids and analgesic were prescribed. Laboratory test showed a white blood cell count of 12 K, Lactate levels were within normal range, and no electrolyte abnormalities were found.

**Fig. 1:**
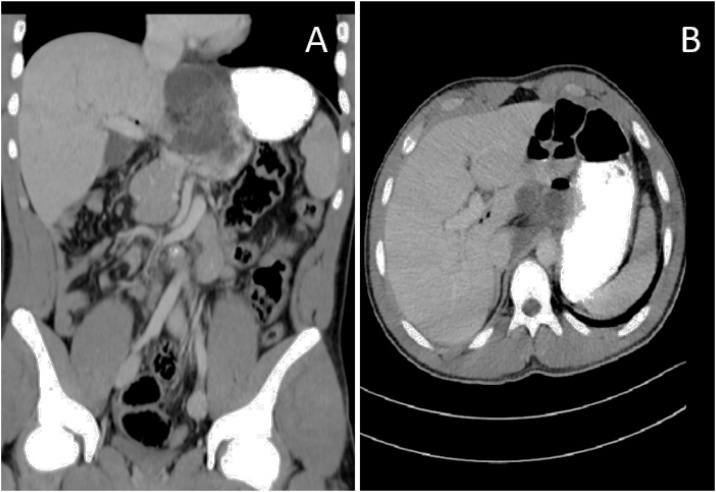
Abdominal computed tomography (CT). (A) Coronal view showing residual contrast material in the stomach, malrotation of the abdomen on the mesenteroaxial axis and air bubbles are found in the falciform ligament. (B) axial section with air bubbles shown in the falciform ligament.

At that point, it was decided to order a CT scan and insert a nasogastric (NG) tube. The NG tube subsequently failed entry. The abdominal CT showed mesenteroaxial gastric volvulus and showed the presence of air within the Falciform Ligament (Fig. 1). Clinical and radiological findings led to the decision to proceed to the operating room. Consultation with a radiologist raised suspicions that possible adhesions were present in the upper abdomen. Furthermore, the patient had a midline laparotomy due to a Ladd's procedure. Consequently, the surgeon, a young specialist 2-year post-surgery residency, felt more comfortable with an exploratory laparotomy rather than a laparoscopic approach.

**Fig. 2:**
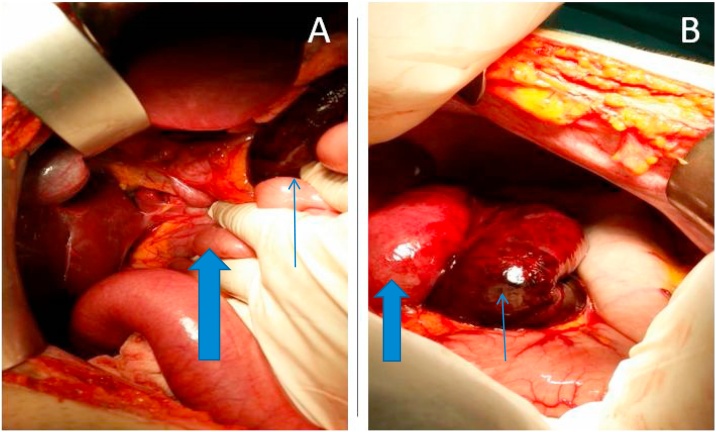
(A) Thick arrow points to the entry point of the jejunal segment to the foramen of Winslow. The thin arrow points to the strangulated loop. (B) Thick arrow marks the distended jejunal loop prior of entry to the foramen of Winslow. The thin arrow points to the strangulated necrotic small bowel loop. The stomach appears normal.

The patient was laid supine, and isoflurane was used for inhaled analgesia. During the operation, the cecum was in lower abdominal cavity and a necrotic strangulated small intestine loop was visualized within the Winslow foramen (Fig. 2). An excision and anastomosis procedure was conducted, and the cecum was fixed in the lower left quadrant.

Postoperatively, the patient felt well and maintained normal vital signs. On the second post-operative day, the abdominal swelling gradually reduced, peristalsis was normal, the abdomen was non-tender and the patient returned to enteral feeding following the ERAS protocol. On post-operative day three, the patient eliminated stool and flatulence. The patient was discharged from the hospital on the fifth post-operative day in good clinical condition with nonrestrictive enteral feeding and minimal abdominal discomfort. The patient was advised to maintain good hydration, avoid weightlifting or straining, use laxatives if required and seek medical attention in case of fever or recurrence of symptoms.

Two weeks after the surgery, during a follow-up at the outpatient clinic, the abdominal pain was completely resolved. He consumed a normal diet, had plenty of fluids and felt well without any further complaints.

## 3 Discussion

Foramen of Winslow hernia was first described by Jacobus Benignus Winslow (1669–1760). It is an extremely rare finding with only 150–200 cases currently described in the literature. The rarity and non-specific presentation, ranging from epigastric abdominal pain through to acute surgical abdomen makes a diagnosis challenging. Moreover, as the incidence of these hernias is most frequent in middle-aged adults, a high degree of suspicion is required when treating a younger patient. Multiple factors have been linked with a foramen of Winslow hernia including an enlarged foramen, a long hypermobile small-intestine mesentery, free mobility of the colon and failure of the colon to enter the retroperitoneal space. However, in this case it appears that a history of a Ladd’s procedure may have had some implication on the subsequent anatomical pathology. So far, it is known that surgical procedures that correct for acquired or congenital abdominal malrotation, such as the Ladd's procedure, have an inherent risk of future recurrent volvulus (2–8 %). However, there is no current literature that can explain the occurrence of a foramen of Winslow hernia after a Ladd's procedure. Therefore, understanding that a Ladd's procedure does lead to at least one predisposing factor for herniation; being hypermobility, makes this working hypothesis and intuitive one. As far as choosing the surgical approach laparoscopic vs laparotomy both seem to be safe in the hand of the skilled and experienced surgeon [9].

## 4 Conclusion

In summary, despite significant improvement in imaging studies, the identification of the foramen of Winslow hernia remains difficult due to its rarity and non-specific presentation. While no conclusions can be made regarding the association of a Ladd’s procedure and a foramen of Winslow hernia, it is recommended to keep such an event on the differential diagnosis.

## Declaration of Competing Interest

All authors declare no conflicts of interest.

## Funding

No funding was received.

## Ethical approval

Exception from ethical approval – case report only, consent from the patient provided at request.

## Consent

The patient provided written, informed consent to the publication of this case report.

## Author contribution

Shadi Abu-Swis, Nir Cohen and Gilbert Sebbag designed the treatment and the protocol. Shadi Abu-Swis, Nir Cohen, Nadav Wallach, Adam Abo-Sharb, Waleed Kian.

Gilbert Sebbag were involved in data collection and writing this manuscript.

## Registration of research studies

1 Name of the registry:
2 Unique identifying number or registration ID:
3 Hyperlink to your specific registration (must be publicly accessible and will be checked):

## Guarantor

Gilbert Sebbag.

## Provenance and peer review

Not commissioned, externally peer reviewed.
